# Can we prevent drug related deaths by training opioid users to recognise and manage overdoses?

**DOI:** 10.1186/1477-7517-6-26

**Published:** 2009-09-25

**Authors:** Romina Lopez Gaston, David Best, Victoria Manning, Ed Day

**Affiliations:** 1 Department of Psychiatry, University of Birmingham, The Barberry Vincent Drive, Birmingham, B15 2FG, UK; 2 Centre for Criminal Justice and Policing, University of the West of Scotland, Hamilton Campus, Almada Building, Almada Street, Hamilton, Lanarkshire, ML3 0JB, UK; 3 National Addiction Centre/Institute of Psychiatry, 1-4 Windsor Walk, Denmark Hill, London, SE5 8AF, UK

## Abstract

**Background:**

Naloxone has been evidenced widely as a means of reducing mortality resulting from opiate overdose, yet its distribution to drug users remains limited. However, it is drug users who are most likely to be available to administer naloxone at the scene and who have been shown to be willing and motivated to deliver this intervention. The current study builds on a national training evaluation in England by assessing 6-month outcome data collected primarily in one of the participating centres.

**Methods:**

Seventy patients with opioid dependence syndrome were trained in the recognition and management of overdoses in Birmingham (n = 66) and London (n = 4), and followed up six months after receiving naloxone. After successful completion of the training, participants received a supply of 400 micrograms of naloxone (in the form of a preloaded syringe) to take home. The study focused on whether participating users still had their naloxone, whether they retained the information, whether they had witnessed an overdose and whether they had naloxone available and were still willing to use it in the event of overdose.

**Results & Discussion:**

The results were mixed - although the majority of drug users had retained the naloxone prescribed to them, and retention of knowledge was very strong in relation to overdose recognition and intervention, most participants did not carry the naloxone with them consistently and consequently it was generally not available if they witnessed an overdose. The paper discusses the reasons for the reluctance to carry naloxone and potential opportunities for how this might be overcome. Future issues around training and support around peer dissemination are also addressed.

**Conclusion:**

Our findings confirm that training of drug users constitutes a valuable resource in the management of opiate overdoses and growth of peer interventions that may not otherwise be recognised or addressed. Obstacles have been identified at individual (transportability, stigma) and at a systems level (police involvement, prescription laws). Training individuals does not seem to be sufficient for these programmes to succeed and a coherent implementation model is necessary.

## Background

Fatal heroin overdose is a significant cause of mortality for injecting drug users (IDUs). Between 1997 and 2002, opiates (including heroin, morphine and methadone) accounted for 6,194 deaths in England and Wales [1]. The mortality rate among opioid drug users is known to be significantly elevated - approximately 2-3% of heroin users die each year and these rates are between six and twenty times higher than those expected among non-drug using peers of the same age and gender [2]. This pattern is found worldwide and in many countries (including the UK) deaths resulting from drug misuse (predominantly opiate overdose) account for as many deaths as road traffic accidents among males [3]. Excess mortality is also well recognised among the sub-population of opiate addicts newly released from prison [4,5]. During the first and second week after discharge, male prisoners were found to be twenty nine times more likely to die, while in females the rate was sixty nine times higher than in the age-matched general population [4].

The majority of opiate-related deaths result from accidental overdose, with at least 50% of opiate users having experienced a non-fatal overdose at some point during their lives [6,7]. Sequelae of non fatal overdose are not rare and represent an additional public health burden [8,9]. Published data has been limited in quantifying the sequelae associated with non fatal overdose. Peripheral neuropathy (resulting from prolonged pressure when unconscious) and pulmonary complications (such as oedema and pneumonia) are the most common complications reported. Rhabdomyolysis accompanied by renal failure and nerve palsy are rare. Cardiovascular complications and cognitive impairments have also been documented. Indirect injuries include physical injuries sustained when falling (while overdosing), burns and assault while unconscious[8].

Research has shown that a high proportion of overdoses are witnessed yet often medical help is not sought or is sought too late [10]. In non-fatal heroin overdoses, emergency services are only contacted on 30-50% [11] of cases, with concerns of police involvement acting as a significant barrier to witnesses accessing emergency services [12]. The presence of bystanders such as peers or family has been seen as an opportunity for intervention in an overdose situation, whilst awaiting the arrival of emergency medical care, based on the recognition that overdose is a process not an event [13]. Harm reduction strategies in this area were first proposed in 1996 to prevent opioid-related deaths through the provision of the opioid antagonist naloxone [14]. These programmes started in Europe, progressed to Australia and the United States where naloxone was first distributed in 1999 through programmes operating in Chicago [15] and San Francisco [16]. Barriers for implementation were noted in areas such as prescription drug laws and drug users' misconceptions about naloxone [12,17].

Naloxone is an opioid antagonist that reverses the effects of opioids in the brain and restores breathing. Its use is associated with transient withdrawal symptoms such as gastro-intestinal disorders, irritability, tachycardia, shivering, sweating and tremor. Most events described in prehospital administration of naloxone are not serious [18,19]. A small but consistent rate of seizures, pulmonary oedema and arrhythmias has been described after postoperative administration. These reports are rare and seemed to be associated with pre-existing cardiac abnormalities and drug interactions, and typically involve significantly higher dose levels than those used in peer overdose interventions [11,20]. Naloxone induced hypertension has also been reported and is possibly related to catecholamine release[20]. Reports from the training programmes have documented life saving events through peer administration without observed side effects, possibly as a result of the lower doses that are typically used in overdose reversal situations [14,21].

Naloxone training and distribution programmes for drug users have provoked controversy among the medical profession and policy makers. Those in favour of issuing naloxone maintain that by training potential witnesses and increasing availability (by relaxing prescription laws) there will be a positive public health impact by reducing the number of drug related deaths among this population [5]. For instance, the study led by Marxwell et al [15] showed a negative correlation between the upward trend of opioid overdose deaths reported by the medical examiner's office and the implementation of the overdose prevention programme in Cook County. Those calling for caution [22,23], maintain that there is a potential for inappropriate use of naloxone by this population with the increased risk of untoward events that could raise issues of liability. So far, inappropriate use of naloxone has not been reported in evaluation studies.

There has been an ongoing debate as to whether the availability of naloxone might promote a 'false sense of security' resulting in a subsequent increase in heroin use. In fact, what limited evidence exists suggests the opposite. Seal et al [16] found that there was a decrease in use of heroin among participants six months after the training; this was attributed to an increase in self efficacy and more insight in relation to personal safety and health obtained during the programme and also resulting from the frightening and aversive effects of witnessing an overdose experienced by the 'rescuer' [15]. Emergency services were found to be contacted less often in those trained in the use of naloxone (10-31%) [11,16,24] in comparison to witnesses of an opioid overdose that did not involve training programmes (30-50%) [11]. This has been associated with fears of arrest [3], an outstanding warrant [11] and increased confidence in reviving the victim [16]; the concern is that these may reduce subsequent engagement with treatment services among overdose victims. However, the majority of studies conducted to date provide little support for the proposed iatrogenic effects of naloxone distribution.

Well designed research into the practice of overdose prevention training and naloxone distribution is limited. Outcomes of established programmes have been measured through the replacement of the naloxone once the supply was used. Studies are subject to self-report bias and lack adequate corroborating evidence. Longitudinal formal evaluation of the cohorts trained has been challenging due to lack of statistical power, high attrition rates, and lack of resources [25,26]. To date only one study has evidenced the effectiveness of overdose training and distribution programmes across different sites in the United States, which despite its limitations has encouraging results. The study compared knowledge about overdose recognition, administration of naloxone and personal competence among groups that only differed on whether they received training on these topics. The study reported that training programmes improve recognition and response to overdoses in the community.

In the current study, we present the results of an evaluation of a cohort of patients followed up for six months after the initial training and immediate supply of naloxone. Training and three month outcomes are described in Strang et al [27], and the current paper extends the evaluation to the 46 patients successfully followed up at the 6-month point, primarily from the Birmingham site but also including the four follow-ups done by the London team. The aim of the study was to assess the effectiveness of training clients in overdose awareness and response, in testing the durability and longevity of acquired knowledge about recognising and intervening in opiate overdose. Additionally, the study assessed whether the clients had retained their naloxone prescription and if so where it was kept and how available it had been in overdose contexts.

## Methods

### Sample characteristics

Between January 2006 and January 2007, 70 patients diagnosed with opioid dependence syndrome were trained in the recognition and management of overdoses in Birmingham (n = 66) and London (n = 4). Out of 70 patients, 65% of the sample was followed up over a 6-month period (n = 46). For details of the training and distribution programme, and the characteristics of the full sample trained see Strang et al (2008). Participants in the cohort were over the age of 18 and had been attending either a detoxification centre or one of six community drug treatment teams at the time of the training session. After the training programme described in Strang et al [27] participants received a supply of 400 micrograms of naloxone (minijet) to take home, on successful completion of the training.

Outreach efforts to recruit participants for the follow-up evaluation included flyers, word of mouth and needle exchange services. Participants were followed up and reinterviewed three months and six months after the training event, if they were available. Interviews were performed over the phone or in face-to-face interviews by one of the authors of the paper (RLG). The interview consisted of a structured questionnaire assessing current use of drugs, whether the trainee had experienced or witnessed an overdose since receiving the supply of naloxone, and if so, what actions were taken. Questionnaires also aimed to measure retention of the knowledge gained during the training on recognition and management of overdoses. Dissemination of information (to relatives/partners/friends) was also tested as well as whether participants were still in possession of the naloxone. On completion of follow up interviews, participants were remunerated with a £10 voucher. Consent was sought from all participants that entered the study.

### Summary of Training Programme

All participants received overdose prevention training by staff (n = 78) that was provided onsite in treatment agencies and the programme involved one of the authors, who prescribed the naloxone on completion of the training (RLG). Opiate users were trained either individually or in small groups (3-10 people) and each training session lasted approximately thirty minutes. Prior to the start of the training a questionnaire was distributed to participants assessing their overdose knowledge and experiences. Overdose training included recognising and discussing the causes of opiate overdose, how to avoid an opiate overdose, signs of an opiate overdose and what to do in this situation. Thus, the initial phase of the training was a harm reduction intervention about overdose recognition and intervention based on placing the individual in the recovery position and calling for an ambulance. The second phase addressed when and how to use naloxone. The naloxone training included information on naloxone, education about appropriate responses to opiate overdose and instructions on naloxone administration.

It was made clear to participants that naloxone was not an alternative to emergency medicine and that an ambulance should be called prior to the administration of naloxone. A dummy of the naloxone minijet was available to demonstrate and practice how to assemble and use the device during the training session. Participants completed post-training questionnaires which were identical to the one given prior to the training, to test changes in knowledge and reported in Strang et al [27]. These questionnaires tested their knowledge about the recognition of overdoses and their management. Upon completion of the overdose prevention training, trainees were issued with one dose of naloxone 400 micrograms minijet with a needle and written information summarizing overdose recognition and revival steps by a doctor (psychiatrist or general practitioner).

### Measurement of knowledge

Participants were asked the same questions at each of four time points - immediately prior to and on completion of the training; at three month follow-up and at 6-month follow-up. These focused on:

*1. Risk factors for overdose *with seven optional answers: (i) using too much heroin, (ii) using heroin alongside other substances, (iii) change in drug purity, (iv) change in tolerance, (v) switching from smoking to injecting heroin, (vi) using heroin alone and (vii) using in unfamiliar places.

*2. Signs of an overdose *with eight optional answers: (i) blood shot eyes, (ii) shallow breathing, (iii) turning blue, (iv) blurred vision, (v) unrousable/loss of consciousness, (vi) fitting, (vii) deep snoring and (viii) pinned pupils.

*3. Actions to take in the event of an overdose *with eleven optional answers: (i) call an ambulance, (ii) stay with the person until they come round, (iii) walk the person around the room, (iv) inject saline solution, (v) give stimulants by mouth, (vi) slap or shake the person, (vii) shock the person with cold water, (viii) perform mouth to mouth resuscitation, (ix) place the person in recovery position, (x) administer naloxone and (xi) stay with the person until the ambulance arrives.

All of the options for risk factors were real risks and so a total score was created out of eight. However, for the other two scales, the options consisted of both correct and incorrect options so the totals represent the number of correct items endorsed (the original questions are included as Appendix 1).

## Results

Seventy participants took part in the study and were trained in recognition and management of overdoses six months prior to the evaluation. Respondents were predominantly male (n = 54, 77%) with a mean age of 34.2 years (± 8.0 years). Of this original sample, 58 people (82.8%) were successfully contacted at the three-month follow-up point and 49 (70.0%) at the 6-month follow-up. However, the sample examined in detail below are those who were interviewed at all three time points (n = 46). This constitutes 65.7% of the cohort originally trained. This group consisted of 35 males and 11 females and had a mean age of 35.0 years.

13 (28.9%) of the group reported that they had ever had an opioid overdose (ranging from 1-6, a total of 34 overdoses in total), while only one person had overdosed in the six months since the initial training. On that occasion naloxone was administered by the ambulance crew and the person had a full recovery. In contrast, nine individuals reported witnessing a total of 16 overdoses in the 6-month period since the training event (range = 1-4 overdoses witnessed). The response to these events is discussed below.

### Knowledge and awareness change following training

#### Indicators of opiate overdose

**Figure 1:**
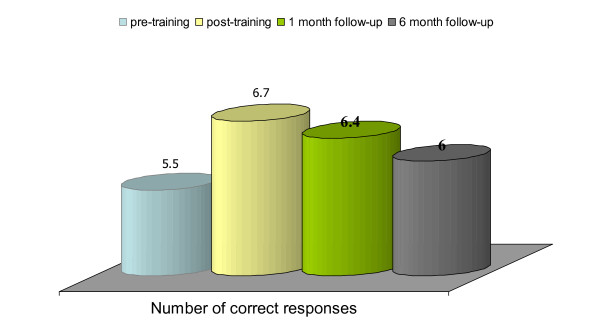
**Changes in recognition across the four time points (pre- to post-training, three months and six months)**.

Figure 1 below shows the change in total scores on accurate reporting of signs of overdose from pre- to post-training and then to follow-up interviews:

In knowledge of signs of overdose, there is a significant improvement from a baseline score of 5.5 out of 7 to 6.7 (t = 5.02, p < 0.001) immediately after the training. In contrast, the reductions in knowledge scores between post-training and three-month follow-up (t = 1.48, p = 0.15), and from three months to six-months post-training follow-up (t = 1.95, p = 0.06) were not statistically significant. There is an overall improvement in average knowledge from baseline (mean = 5.5 out of 7) to follow-up (mean = 6.0 out of 7) that is statistically significant (t = 2.25, p < 0.05) suggesting that knowledge of overdose signs is retained over time.

#### Actions to take in overdose events

**Figure 2:**
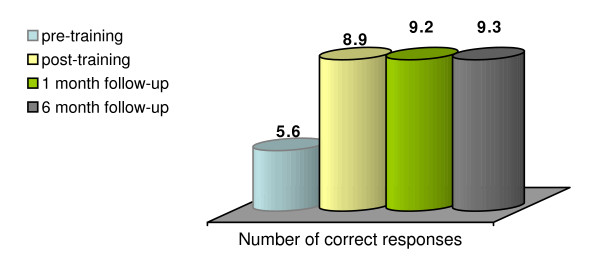
**Changes in actions to be taken from pre to post-training and in each follow-up for actions in response to overdose**.

As shown in Figure 2 below, there is a similar improvement in knowledge of actions to be taken.

There was a significant increase in the number of appropriate actions to taken identified from pre-training to post-training (5.6 to 8.9 out of 11; t = 7.60, p < 0.001). There were further (non-significant) increases in the average correct scores from post-training to three month follow-up (mean score of 8.9 to 9.2; t = 0.67, p = 0.51), and again from three months follow-up to six months (mean increase from 9.2 to 9.3; t = 1.03, p = 0.31). Overall, there was a marked increase in knowledge from baseline to 6-months (from a mean score of 5.6 to 9.3, t = 9.62, p < 0.001). Thus, across the two scales measured at both time points, clients showed consistently improved levels of knowledge.

#### Naloxone possession and retention

At the three month follow-up, 40 of the 46 clients (87.0%) reported that they still had the naloxone that they were given at the end of the training session. Of the remaining, 2 reported that they had lost it and 4 were not sure. At the six-month follow-up, 37 of the 46 participants still had the naloxone (80.4%), 3 had lost it, one had thrown it away because the minijet had passed its 'expiry date', one reported that it had broken, one returned it to their treatment worker when they stopped using heroin, and one had thrown it away when they started inpatient detoxification treatment. The data for the other two cases were missing. However, of the 37 people who retained their naloxone, seven did not keep it at home - thus for 30 of 37 clients (81.0%), the naloxone could only be used if the overdose occurred in their own home.

Although no differences in pre-training knowledge, those who still had their naloxone at the six-month follow-up point, reported significantly higher mean post-training knowledge of signs indicative of opioid overdose (see Table 1):

**Table 1:** Knowledge as a predictor of naloxone retention

	**Lost (n = 9)**	**Retained (n = 37)**	**T, sig**
Post - risks	4.9	6.1	1.25

Post - signs	5.3	7.1	3.78***

Post - actions	7.1	9.3	1.68

Clients who had higher knowledge scores after the training were more likely to still have the naloxone minijet 6 months later, and this difference was significant for their knowledge of overdose signs. In total, 16 clients reported that they trained others in how to use naloxone, but this was not related to their own knowledge or awareness.

#### What happened in the event of overdose after the training?

As indicated above, a total of nine individuals reported that they witnessed 16 overdoses in the 6 month period after the training. Seven of the nine people who witnessed overdoses reported that they still possessed their naloxone at the time of the witnessed overdose, of whom four reported that they kept it at home, two in their bag and for one case this information was missing. The reasons for non-use were not related to failing to recognise that an overdose was taking place - all 9 reported that they felt confident that they would recognise an overdose. The following responses were given as indicators of overdose at the time:

• shallow breathing (4/9)

• blue lips (5/9)

• pinned pupils (2/9)

• unresponsive to pain (1/9)

• unconscious (5/9)

In relation to the actions taken during the witnessed overdoses, none of the individuals reported taking any inappropriate action that could have endanger the victim's situation (e.g. walking the person around the room, injection of saline solution, administration of oral fluids, putting the person in a bath). Actions taken during the overdose were in agreement with the training received, for instance witnesses:

• Called an ambulance (3/9)

• Placed the person in the recovery position (2/9)

• Stayed with the person until they came round (2/9)

• Stayed with the person until the ambulance arrived (2/9)

• Checked airways for obstruction (3/9)

• Checked breathing (4/9)

• Performed mouth to mouth resuscitation (2/9)

• Checked the pulse (3/9)

Out of the 16 people who had overdoses that were witnessed by participants in the study, one was already dead when found, six survived and data was missing for the rest. From those who survived, naloxone was used in three cases by the ambulance crew with no reports of adverse reactions and two individuals were admitted to hospital. From those that witnessed overdoses, five did not use their supply of naloxone, and data is missing from the other four cases. In other words, in those five cases in which data is available, none used the naloxone prescribed after the training. The reasons given for this were:

• Naloxone was lost (1/5)

• Not wanted to be found with injecting equipment in place of work (1/5)

• Person was 'clean' (no longer using illicit substances) and did not want to carry injecting material (2/5)

• Not appropriate as person already dead when found (1/5)

• Data missing (4/9)

## Discussion

The results of this study suggest that training opiate users in the recognition and management of opiate overdoses has a significant impact on their awareness, knowledge and confidence, and increased their likelihood to intervene in high risk situations. In areas such as identification of risk factors/signs of opiate overdose, and the knowledge of appropriate actions that need to take place, the comparison of pre-training scores and scores six months after the training demonstrates consistent retention of knowledge, with only slight deterioration in awareness of signs although these remained above the baseline level. The improvements in knowledge post-training for appropriate actions could be related with the rehearsal and consolidation of information that had taken place in each of the follow up points throughout the study; showing potential opportunities for refresher courses in the target population after the initial training.

In addition, the majority of individuals trained still possessed the naloxone six months later suggesting a commitment to the process of peer education and intervention. It is intriguing to note that knowledge reported at the end of the training appeared to predict whether people will retain the naloxone, suggesting that those clearest about when and how to use naloxone are also those who are most likely to retain the minijet. While most overdoses occur in residential settings [10], we cannot assume that this is always the home of the person to whom the naloxone is prescribed. Thus, the transportability of the naloxone and the willingness of the recipient to carry it are key to the success of naloxone distribution schemes. In our study, most of the individuals that kept naloxone did so at home, and from those witnesses for whom information is available, none of them was in possession of the medication when the overdose occurred. This appears to contradict the reported willingness to use naloxone reported in the earlier London study [28]. Two reasons for the reluctance to carry naloxone are perceived stigma and fear of police engagement, and the awkwardness of carrying something bulky and unwieldy. It would be anticipated that improvements in product development supplemented by increased awareness of naloxone programmes in target areas would break down some of these barriers to trainees carrying their naloxone. The data available from some of the witnesses suggest that they wouldn't carry naloxone with them due to issues related with stigma (not wanting to be found with injecting material if searched, the association between injecting material and using illicit drugs, etc) and their drug taking status (being 'clean' or in recovery as opposed to actively using illicit substances). This may suggest that willingness alone is not sufficient for this intervention and users have to be confident that the police and ambulance services will not have detrimental reactions to them having naloxone. An additional factor that could have biased the results in this direction could be related to the recruitment of the cohort. Participants were recruited exclusively from treatment settings and those followed up were mostly patients discharged after residential opioid detoxification. Issues related with stigma in carrying naloxone in this population could have been enhanced by a perceived conflict between their recovery pathway after detoxification - and moving away from drug-using peers - and the 'conflicting' desire to carry a medication in the event of witnessing an opioid overdose situation. While this is in practice a good location in which to access and train drug users, their own abstinence-oriented treatment plans may be a barrier to successful intervention and to their willingness to carry naloxone. The use of treatment populations generally present different challenges in understanding the scope for naloxone use by peers that are partly shaped by the social networks of treated clients and their levels of ongoing exposure to drug use. An important development in our knowledge of naloxone utility will be to understand the relative impact of programmes that target in treatment compared to out of treatment populations of heroin users.

In terms of formulation and type of prescription, the relatively bulky mechanism of a minijet may make this unattractive to users and the appearance of a needle may be a psychological barrier to former users who have stopped using. Further investigation of other options, such as nasal sprays or more discrete presentations may be beneficial in overcoming these barriers to naloxone availability. There have been trials in which intranasal naloxone was used as first line intervention in prehostpital setting [29,30]. Evidence is still lacking in relation to the effectiveness, safety and utility of this route of administration for naloxone [5]. Most crucially, user group involvement in the dissemination process may assist in addressing each of these concerns.

The importance of peer group work is emphasised by the findings around 'secondary training'. A third of the sample reported that they had trained significant others in overdose recognition and management, this being an important element in the chain of knowledge triggered by the study, and which is informing the current work we are doing which involves peers in the delivery of the initial training package. Previous studies [12,17] suggest that issues related to the presence of the police would deter individuals from contacting the ambulance services as part of the actions taken when facing an overdose situation. This study was not designed to elicit this particular aspect; however, the available data suggests that from the 16 overdoses witnessed, police presence was reported in one occasion after contacting emergency services. It is critical that both the reality of police involvement is addressed through inter-agency working and that the perception of police involvement in overdose is also addressed through treatment services and user involvement groups.

The study is limited by the small sample size, recruitment biases, missing data and the problems associated with study attrition. It is not known what the rates of knowledge or naloxone retention were in the group that could not be contacted for this study, and the use of primarily one location means that there may also be local effects relating to the nature of the training and the group accessed in this one location, a UK city with a low rate of intravenous drug use. Similarly, the study is entirely reliant on self-report and we have not been able to corroborate the reports around the witnessed overdose events reported. Accessing trainees after the event has proved to be difficult and we had to rely on brief phone conversations in some cases, resulting in large amounts of missing information from a few participants.

In summary, our findings confirm previous reports that the training of possible bystanders to opiate overdose constitutes a valuable resource in the assessment and management of opiate overdoses that may not otherwise be recognised or addressed. This has been demonstrated by the increased levels of knowledge retention associated with high confidence and willingness to keep the medication six months after the training took place. Obstacles have been identified at individual and at a systemic level. For instance, there are issues of transportability of naloxone related to its formulation and also perceived stigma (the association of this drug with the 'active user of illicit substances' status). This is related with overpowering fears of being searched by the police whilst in possession of naloxone, as well as police involvement when the emergency services are contacted. Witnesses' concerns of being treated as responsible parties if naloxone is used at the scene when an overdose takes place, have been reduced by education about prescription laws during the training. The reclassification of naloxone under article 7 of Prescription Only Medicines Order in the UK, allows the administration of naloxone by injection by anyone for the purpose of saving a life in an emergency. However UK laws still hold naloxone as a prescription medication that requires a face to face encounter for the medication to be legally prescribed on a 'patient named bases'. As stated above, a third of the sample trained significant others in the recognition and management of an opiate overdose. According to current prescription laws this subpopulation cannot be provided directly with naloxone. Innovative training schemes [31] have trained opioid users with significant others ('buddies') increasing the opportunities to prescribe directly to patients with the involvement of those that care for them (potential witnesses). This strategy elegantly uses the current legal framework as a bridge rather than hindrance towards naloxone distribution by prescribing to the patient accounting for emergency use by the significant other. Whilst this paper is being written, the UK government started to launch a pilot scheme through the National Treatment Agency [32] by which this practice is being encouraged countrywide.

Consequently, training individuals does not seem to be sufficient for these programmes to succeed and a more systemic approach is necessary. Changes in prescription laws, increasing education and communication between the police force, emergency services and opiate users and reducing the stigma that prevails in these areas, are essential ingredients for these programmes to move forward. The complexities of these changes mean that existing schemes should be innovative and in constant development to progress within the current constraints.

## Competing interests

The authors declare that they have no competing interests.

## Authors' contributions

RLG participated in the sequence, alignment and drafted the manuscript. DB participated in the sequence, alignment and performed the statistical analysis, VM participated in the statistical analysis and draft of the manuscript and. ED helped to draft the manuscript. All authors read and approved the final manuscript.
